# Influence of exogenous growth hormone administration on circulating concentrations of α-klotho in healthy and chronic kidney disease subjects: a prospective, single-center open case-control pilot study

**DOI:** 10.1186/s12882-018-1114-z

**Published:** 2018-11-15

**Authors:** Aaltje Y. Adema, Camiel L. M. de Roij van Zuijdewijn, Joost G. Hoenderop, Martin H. de Borst, Piet M. Ter Wee, Annemieke C. Heijboer, Marc G. Vervloet, for the NIGRAM consortium,, R. Bindels, J. G. Hoenderop, M. H. de Borst, J. L. Hillebrands, G. J. Navis, P. M. ter Wee, M. G. Vervloet

**Affiliations:** 1 Department of Nephrology, VU University Medical Center, De Boelelaan 1117, 1081 HV Amsterdam, The Netherlands; 2 Department of Physiology, Radboud University Medical Center Nijmegen, Nijmegen, The Netherlands; 3 Department of Internal Medicine, Division of Nephrology, University Medical Center Groningen, Groningen, The Netherlands; 4 Department of Clinical Chemistry, VU University Medical Center, Amsterdam, The Netherlands; 5 Amsterdam Cardiovascular Sciences (ACS), Amsterdam, The Netherlands

**Keywords:** α-Klotho, Growth hormone, Chronic kidney disease

## Abstract

**Background:**

The CKD-associated decline in soluble α-Klotho (α-Klotho) levels is considered detrimental. Some studies suggest a direct induction of α-Klotho concentrations by growth hormone (GH). In the present study, the effect of exogenous GH administration on α-Klotho concentrations in a clinical cohort with mild chronic kidney disease (CKD) and healthy subjects was studied.

**Methods:**

A prospective, single-center open case-control pilot study was performed involving 8 patients with mild CKD and 8 healthy controls matched for age and sex. All participants received subcutaneous GH injections (Genotropin®, 20 mcg/kg/day) for 7 consecutive days. α-Klotho concentrations were measured at baseline, after 7 days of therapy and 1 week after the intervention was stopped.

**Results:**

α-Klotho concentrations were not different between CKD-patients and healthy controls at baseline (554 (388–659) vs. 547 (421–711) pg/mL, *P* = 0.38). Overall, GH therapy increased α-Klotho concentrations from 554 (405–659) to 645 (516–754) pg/mL, *P* < 0.05). This was accompanied by an increase of IGF-1 concentrations from 26.8 ± 5.0 nmol/L to 61.7 ± 17.7 nmol/L (*P* < 0.05). GH therapy induced a trend toward increased α-Klotho concentrations both in the CKD group (554 (388–659) to 591 (358–742) pg/mL (*P* = 0.19)) and the healthy controls (547 (421–711) pg/mL to 654 (538–754) pg/mL (*P* = 0.13)). The change in α-Klotho concentration was not different for both groups (*P* for interaction = 0.71). α-Klotho concentrations returned to baseline levels within one week after the treatment (*P* < 0.05).

**Conclusions:**

GH therapy increases α-Klotho concentrations in subjects with normal renal function or stage 3 CKD. A larger follow-up study is needed to determine whether the effect size is different between both groups or in patients with more severe CKD.

**Trial registration:**

This trial is registered in EudraCT (2013–003354-24).

**Electronic supplementary material:**

The online version of this article (10.1186/s12882-018-1114-z) contains supplementary material, which is available to authorized users.

## Background

The excessively high cardiovascular (CV) risk in patients with chronic kidney disease (CKD) is only partially explained by the higher prevalence of traditional risk factors [1]. Therefore, other CKD-related factors are believed to play a causal role, such as deregulation of the fibroblast growth factor 23 (FGF23)-Klotho-vitamin D axis [2]. The anti-aging α-Klotho protein was discovered in 1997 following manipulation of its gene [3]. α-Klotho is predominantly synthesized in the distal tubular epithelial cells of the kidneys and in lower levels in the proximal tubule [4]. The extracellular domain is cleaved and released into extracellular fluid, including blood, cerebrospinal fluid and urine [3]. As CKD progresses, α-Klotho concentrations decrease [5]. Lower α-Klotho concentrations are associated with progressive CKD [5], higher prevalence of cardiovascular disease [6], arterial stiffness [7] and vascular calcification [8]. Animal studies showed that restoration of α-Klotho reduces oxidative stress, attenuates hypertension, ameliorates cardiac hypertrophy and prevents endothelial dysfunction [9–12]. Therefore, increasing α-Klotho concentrations may be a legitimate goal in CKD patients in order to slow down or even reverse these processes. However, clinical long-term exogenous supplementation of the relatively large α-Klotho-protein (130 kDa) might be an option for the far future in human and therefore upregulation of the endogenous production of α-Klotho might be more feasible, at least in the predialysis phase, as the kidney is the primary production site of α-Klotho. Several recent studies assessed different experimental options to up-regulate endogenous α-Klotho [13–21]. In humans, the use of angiotensin-receptor blockers (ARBs) and vitamin D were shown to increase α -Klotho concentrations to some extend [21, 22]. However, despite the widespread use of vitamin D en ARBs in patients with CKD, the frequency of CV events and mortality in patients with CKD remains high. Recent data showed a complex relationship between growth hormone (GH) and α-Klotho concentrations [23]. Whether IGF-1 or GH directly affects α-Klotho concentrations is still unknown, although small pilot studies showed that GH replacement therapy in both children and adults with GH deficiency increased α-Klotho concentrations [24, 25]. However, the effect of administration of exogenous GH on the α-Klotho concentration in subjects with CKD and healthy controls is unknown.

In the present study, the effect of subcutaneous GH therapy on α-Klotho concentrations in subjects with or without mild CKD is investigated in a prospective, single-center open-label case-control pilot study.

## Methods

### Participants and intervention

In total, 18 subjects (12 men and 6 women) with or without CKD stage 3 (creatinine clearance of 30–60 mL/min/1.73m^2^ according to the Chronic Kidney Disease Epidemiology Collaboration (CKD-EPI)) were included in the period of January 2015 until March 2016 from the outpatient clinic of nephrology in the VU medical center. Subjects were matched for age and sex, to allow an adequate comparison between those with and without CKD. Exclusion criteria were the use of immunosuppressive agents, GH suppletion, oestrogens, corticosteroids, androgens, or anabolic steroids. Furthermore, subjects with any pituitary disease, history of malignancy, respiratory disorder or obstructive sleep apnoea syndrome, known thyroidal disease, active vasculitis, heart failure, severe hepatic disease, chronic systemic infections, uncontrolled hypertension, diabetes mellitus, malnutrition, autosomal dominant polycystic kidney disease, single kidney or a BMI > 30 kg/m^2^ were also excluded. All included subjects received subcutaneous GH injections (Genotropin®, 20 mcg/kg/day) for 7 consecutive days. The primary end point was the change in α-Klotho concentrations after 7 days of GH-administration. Secondary endpoint was the potential difference in change of α-Klotho concentration between patients with CKD and healthy subjects.

### Assays

Non-fasting blood samples and first morning spot urine were drawn at baseline, after 7 days of treatment and 1 week after the treatment stopped. Collected material was stored at − 80 °C until use. No additional freeze-thaw cycles were needed. IGF-1 was measured in serum samples using an immunochemiluminescent assay (Liaison, DiaSorin®). Concentrations of creatinine, phosphate, C-reactive protein, glucose, albumin and calcium were measured in heparin samples (Cobas, Roche Diagnostics). Urine creatinine, calcium, phosphate and albumin were measured in first morning spot urine samples (Cobas, Roche Diagnostics). Fractional excretion of phosphate was calculated using spot urine samples. α-Klotho was measured in − 80 °C stored heparin samples using a α-Klotho immunoassay (IBL international GmbH, Hamburg, Germany) with an intra-assay variation of < 5% and an inter assay variation < 7.5% [26]. C-terminal FGF23 was measured in EDTA-plasma using ELISA (Immutopics) [27] with an intra-assay variation of < 5% and an inter assay variation < 10%. Tubular maximal reabsorption of phosphate normalized to GFR (TmP/GFR) was used as an index of the renal threshold for phosphate excretion, calculated from values in serum and spot urine according to the nomogram by Walton and Bijvoet [28].

### Statistical analysis

Baseline characteristics are shown as mean (standard deviation), median (interquartile range (IQR)) or number (percentage), when appropriate. Normally distributed numerical variables were compared using an unpaired T-test, non-parametric data with a Mann-Whitney U test and categorical variables by a Chi-square test. Longitudinal data were analysed with linear mixed models (LMM) with a random intercept, a random slope or both, based on the lowest Aikaike’s Information Criterion. For all analyses, an autoregressive covariance matrix was used. All model assumptions were checked and not violated. To test whether the effect of growth hormone administration on α-Klotho was different for CKD patients or healthy controls, a LMM was fitted with an interaction term between time and group. A *p*-value < 0.05 was considered statistically significant. All analyses were performed using IBM SPSS Statistics software version 20 (IBM Inc., IL, USA) (Additional file 1).

## Results

### Characteristics study population

**Fig. 1:**
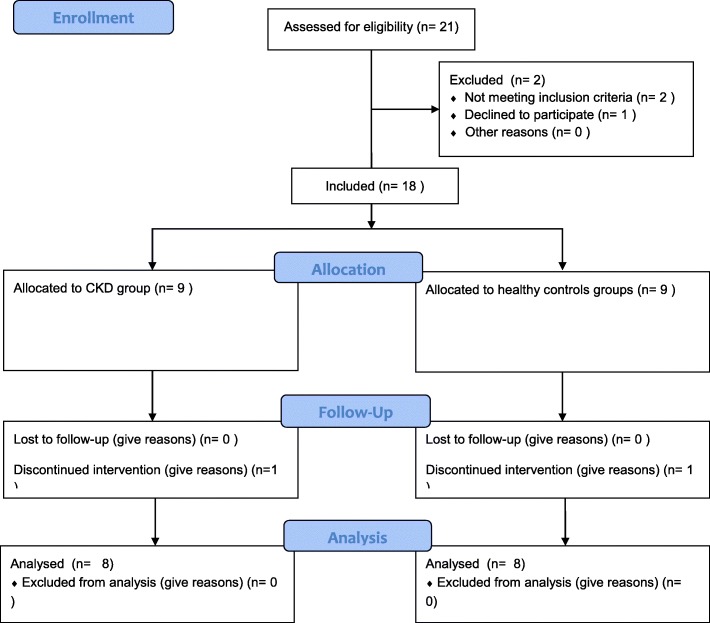
CONSORT Flow Diagram

All subjects, except one tolerated the administration of GH well. One male subject in the CKD subgroup discontinued the study due to complaints of headache. Furthermore, 1 male subject in the healthy control subgroup was withdrawn due to a serious adverse event (SAE) during the study. This SAE, a hospital admission for pain and acute kidney injury due to an obstructive kidney stone, was not related to study procedures. Thus, data on 16 subjects were analysed, 8 patients in the CKD-group and 8 in the healthy control group. This study adheres to the CONSORT guidelines (Fig. 1). Mean age of the participants was 46 years old (ranging from 25 to 59 years old). Mean eGFR in the CKD-subgroup was 57 ± 17 mL/min/1.73 m^2^). As can be seen in Table 1, baseline characteristics are comparable between the two groups, except for eGFR by definition of the groups.

**Table 1:** Baseline characteristics of the participants^a^

	CKD stage III (*n* = 8)	Healthy controls (n = 8)	*p* for difference
Age (years)	46.9 ± 12.9	44.5 ± 11.4	0.70
Male, no. (%)	5 (62.5)	5 (62.5)	1.00
BMI (kg/m^2^)	23.5 ± 2.8	25.3 ± 2.9	0.23
Smokers, no. (%)	1 (12.5)	0 (0)	0.30
SBP (mmHg)	134 ± 13	133 ± 10	0.87
DBP (mmHg)	82 ± 11	78 ± 6	0.33
eGFR# (ml/min/1.73 m2)	57 ± 17	100 ± 8	< 0.01
IGF-1 (nmol/L)	26.3 ± 2.8	27.3 ± 6.8	0.71
Serum phosphate (mmol/L)	0.89 ± 0.16	1.01 ± 0.16	0.16
PTH (pmol/L)	7.3 ± 3.1	4.7 ± 1.2	0.05
25(OH)D3 (nmol/L)	70 ± 20	76 ± 30	0.69
cFGF23 (RU/mL) (median + IQR)	100 (77–127)	92 (80–105)	0.57
CRP < 10 (mg/L)	8 (100%)	8 (100%)	n/a
Albumin (g/L)	38.3 ± 2.1	38.0 ± 2.3	0.82
α-Klotho (pg/mL) (median + IQR)	554 (388–659)	547 (421–711)	0.57

^a^Values are expressed as mean ± SD, unless specified otherwise. *IQR* interquartile range

^b^Estimated GFR expressed using the Chronic Kidney Disease Epidemiology Collaboration (CKD-EPI) equation

### IGF-1 concentrations

After 7 days of GH suppletion therapy (GHST), IGF-1 concentrations, as indicator of GH therapy bioactivity, increased from 26.8 ± 5.0 nmol/L to 61.7 ± 17.7 nmol/L (*P* < 0.05). Mean IGF-1 concentrations increased from 26.3 ± 2.8 nmol/L to 59.8 ± 20.5 nmol/L (*P* < 0.05) and from 27.3 ± 6.8 nmol/L to 63.6 ± 15.6 nmol/L (*P* < 0.05) in the CKD-group and healthy controls respectively. The increase in IGF-1 concentrations was not different over time between the CKD subgroup and the healthy controls, (P for interaction = 0.71, Table 2).

**Table 2:** Time-related results within and between groups

	Entire cohort		Patients with CKD		Healthy controls		*P* interaction (time*group)
	Absolute change after 1 week of growth hormone administration (95% CI)	*P*	Absolute change after 1 week of growth hormone administration (95% CI)	P	Absolute change after 1 week of growth hormone administration (95% CI)	P	
IGF-1 (nmol/L)	34.9 (27.5–42.3)	< 0.01	33.5 (21.8–45.2)	< 0.01	36.4 (25.4–47.3)	< 0.01	0.71
Phosphate (mmol/L)	0.04 (−0.04–0.12)	0.34	−0.02 (− 0.16–0.12)	0.78	0.10 (0.00–0.19)	0.05	0.15
Urinary phosphate excretion (mmol/L)	4.94 (−3.73–13.62)	0.25	−2.99 (−9.56–3.58)	0.34	12.88 (− 3.10–28.9)	0.11	0.06
TMP/GFR (mmol/L)	0.06 (−0.04–0.17)	0.22	0.01 (−0.09–0.12)	0.78	0.11 (− 0.08–0.30)	0.23	0.35
PTH (pmol/L)	−0.19 (−1.09–0.70)	0.66	− 0.94 (−2.64–0.77)	0.26	0.55 (− 0.18–1.28)	0.13	0.10
cFGF23 (RU/mL)	26.1 (15.7–36.6)	< 0.01	27.9 (12.3–43.5)	0.01	24.4 (8.0–40.8)	0.01	0.74
α-Klotho (pg/mL)	81.1 (1.7–160.4)	0.05	96.4 (−52.2–245.0)	0.19	65.8 (−20.8–152.3)	0.13	0.71

95%CI = 95% confidence interval. P interaction (time*group) is the interaction term between the CKD group and healthy controls

### Effect of subcutaneous growth hormone therapy on circulating α-klotho concentrations

**Fig. 2:**
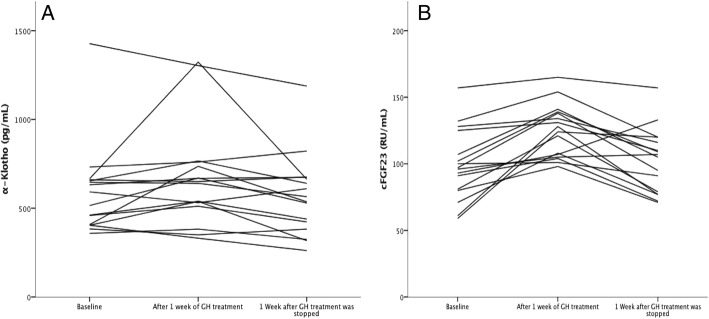
Serum α-Klotho and cFGF23 concentrations of the subjects per visit

At baseline, α-Klotho concentrations were not statistically significant different between CKD-patients and healthy controls (Table 1, *p* = 0.38). Median α-Klotho concentrations increased from 554 (IQR 405–659) to 645 (IQR 516–754) pg/mL (*P* = 0.05). As can be seen by Fig. 2a, the variability in response is rather high. α-Klotho concentrations increased from 554 (IQR 388–659) to 591 (IQR 358–742) pg/mL (*P* = 0.19) and from 547 (IQR 421–711) pg/mL to 654 (IQR 538–754) pg/mL (*P* = 0.13) in the CKD and the healthy subgroup respectively. The difference in change of α-Klotho concentration was not statistically significant between the two subgroups (*p* for interaction = 0.71). All α-Klotho concentrations returned to baseline levels within one week after the treatment being stopped (Fig. 2a).

### Figure 2: The effect of endogenous growth hormone therapy on serum α-klotho and cFGF23 concentrations

#### Serum cFGF23, serum phosphate, urinary phosphate excretion, TmP/GFR and PTH

Median of cFGF23 changed from 96.5 RU/mL (IQR: 80.3–120.5) to 126.0 RU/mL (IQR: 105.5–138.8; *p* < 0.05, Fig. 2b). In the CKD subgroup, median cFGF23 changed from 99.5 RU/mL (IQR: 77.3–127.3) to 132.5 (IQR: 112.0–138.8) (*P* < 0.05) and in healthy controls from 92.0 RU/mL (IQR: 80.3–105.3) to 114.0 RU/mL (IQR: 101.8–137.8) (P < 0.05). The rate of change in cFGF23 concentrations was not different between the two subgroups (P for interaction = 0.74, Table 2).

Serum phosphate concentrations, urinary phosphate excretion, the TmP/GFR and PTH did not change significantly in the entire cohort or both individual groups (Table 2).

## Discussion

The main finding of our study is that GH therapy increases serum α-Klotho concentrations in subjects with normal kidney function or stage 3 CKD. α-Klotho concentrations increased in both subgroups, although within subgroups the increase did not reach statistical significance, most likely due to small subgroup size.

These results are in line with previous studies showing that GH therapy increases α-Klotho concentrations in GH deficient, paediatric and adult patients [24, 25]. Although the increment of α-Klotho concentrations was more prominent in the small study group of Locher et al.. However, they included GH-deficient subjects whereas in the present study GH-sufficient subjects were included. It is conceivable that an additional increment of α-Klotho concentrations is more difficult to achieve if IGF-1 concentrations are already sufficient.

Previous studies have convincingly shown that α-Klotho concentrations decrease as kidney function declines [29]. However, both α-Klotho and FGF23 concentrations in our patients of the CKD subgroup, which are classified as mild-moderate CKD according to the CKD-EPI were not significantly different from the healthy controls at baseline. This underlines the literature that shows that eGFR loss and decrease of serum α-Klotho concentrations do not parallel [30], and may depend on the ELISA used [26]. Moreover, there is oversampling in the CKD-subgroup close to stage 2 CKD, where soluble α-Klotho concentrations may be maintained in the normal range. Importantly, our study was underpowered to make firm statements about differences between the two subgroups.

Our findings show that α-Klotho concentrations are modifiable using administration of exogenous GH in a clinical cohort of subjects with mild CKD and healthy subjects. This increase may be of clinical relevance for patients with CKD in terms of CKD progression and cardiovascular risk as animal studies show that even small increases in α-Klotho concentrations are protective for remnant kidney function and attenuates cardiovascular intermediate endpoints [13, 31–33]. Obviously, this concept requires clinical studies to be confirmed.

Despite the reduced bioactivity of GH and IGF-1 observed in CKD, there is a valid rationale for the use of GH in this setting. Indeed, treatment with GH results in a decrease of serum IGFBP-1 concentrations and a marked increase in serum insulin, IGF-1, IGFBP-3 and IGFBP-5 concentrations, which subsequently leads to a marked increase in IGF-1 bioactivity [34, 35]. In a previous study exogenous GH therapy had no effect on all-cause mortality and cardiovascular morbidity and mortality in haemodialysis patients [36]. Although at that time its possible effect on α-Klotho was unknown. It is unlikely that major increments of α-Klotho did occur in these patients with end-stage kidney disease as the kidneys are the principal source of α-Klotho [37]. Moreover, the study was terminated early, none of the subjects completed the study and follow-up was short. On the contrary, some small short-term studies tested the effect of GH therapy in earlier stages of CKD and noted that GH therapy significantly improved LDL-cholesterol, phosphate and capillary blood flow, however no significant effect was demonstrated on intermediate endpoints, namely total peripheral vascular resistance and cardiac output [38, 39]. It would be very interesting to apply GH suppletion in well-powered studies including patients with CKD stage 4 and 5, not on dialysis, as well.

The absolute increase in α-Klotho concentrations in our study population was modest. This is also exemplified by the lack of robust effect on phosphate homeostatic parameters, measured in our study, including serum phosphate concentration and urinary excretion. The study design however precludes concluding if this effect would have been stronger with a longer duration or a higher dose of administrated GH. Given the strong phenotypic similarity between α-Klotho knockout models and CKD, and the wide range of CKD-related pathologies that in experimental studies can be attenuated by exogenous α-Klotho, additional exploration is warranted of all options that upregulate endogenous α-Klotho, including GH therapy.

In agreement with other studies, our study showed that cFGF23 increases after GH therapy [25, 40]. However, previous studies also reported an increase in serum phosphate concentrations, which was not observed in the present study. Therefore, the hypothesis from the earlier studies that GH therapy induces FGF23 production in response to increased serum phosphate concentrations is not confirmed in our study [25]. Besides a stable serum phosphate concentration, phosphate excretion did not change either, despite an increase in cFGF23 and a slight increase in α-Klotho level. The explanation for a lack of effect on renal phosphate handling is not obvious from our data, although one could speculate that GH induced cleavage of tubular α-Klotho concentrations, leaving tubular cell deprived of α-Klotho, and as such promoting FGF23 resistance. Data on the effect of GH and IGF-1 on serum phosphate concentrations are highly contradictory [41, 42]. Unfortunately, only cFGF23 was measured in this study. However, the study of Effthymiadou et al. in 23 children with a GH-deficiency showed that both cFGF23 and iFGF23 increase after GH administration [25].

Bianda et al. reported a significant increase of serum 1,25-dihydroxyvitamin D3 (1,25-(OH)_2_D_3_) concentrations after GH- or IGF-1 therapy [41]. Serum 1,25-(OH)_2_D_3_ is known to upregulate FGF23 gene expression in bone and consequent gives a rise in serum FGF23 concentrations [43–47]. Therefore, the observed increase of serum cFGF23 concentrations might be explained by an assumed GH-induced rise in serum 1,25-(OH)_2_D_3_ levels. Unfortunately, vitamin D concentrations were measured only at baseline in this study. Moreover, IGF-1 and GH treatment increase markers of bone turnover like serum osteocalcin and carboxyterminal propeptide of type 1 procollagen (PICP) as indicators of osteoblast activity [41, 42]. Therefore, it is conceivable that GH has an indirect effect through IGF-1 on bone turnover and osteoblasts, one of the cell types, besides osteocytes, that produce FGF23. It is unknown if the potential beneficial effects of an increase of α-Klotho concentrations can outweigh the assumed dismal effects of increased cFGF23 concentrations.

Besides the small sample size of this study, there are some other limitations that need to be underlined. First, the exclusion criteria for participants limit generalizability, in particular for patients with more advanced CKD. Second, the specificity of the IBL-assay used to measure α-Klotho concentration is disputed [26, 48]. We did not use the semi-quantitative precipitation-immunoblotting technique as described by Barker et al., which probably has improved specificity [29]. This method awaits external validation in a different cohort and by different laboratories. Moreover, we recently found that the ELISA used in our study performs best among currently commercially available immunoassays [26]*.* Unfortunately, we were not able to assess the influence of GH therapy on membrane-bound α-Klotho due to the absence of kidney biopsies in our study. Finally, a study of longer duration is needed to determine the more long-term effects of GH on α-Klotho concentrations in the CKD population, and establish a dose-response effect. Our study however was designed as a proof of concept to study the modifiability of α-Klotho by GH.

## Conclusions

In conclusion, exogenous GH therapy can induce a significant increase in α-Klotho concentrations in subjects with normal kidney function or stage 3 CKD. It is unknown if this can also be accomplished in more advanced CKD. Additional studies are necessary to study whether this increase of α-Klotho concentrations improves intermediate endpoints and subsequently patient-level outcome.

## Additional file

Additional file 1: **S1** Final fulle database K&G study. This database contains the datasets used and/or analysed during the current study. (XLS 80 kb)

## Abbreviations

ARBs: Angiotensin-receptor blockers
BMI: Body mass index
CKD: Chronic kidney disease
CKD-EPI: Chronic Kidney Disease Epidemiology Collaboration
CV: Cardiovascular
eGFR: Estimated glomerular filtration rate
FGF23: Fibroblast-growth-facor-23
GH: Growth hormone
GHST: Growth hormone suppletion therapy
IGF-1: Insulin growth factor-1
IQR: Interquartile range
LMM: Linear mixed models
PTH: Parathyroid hormone
SAE: Serious adverse event
TmP/GFR: Tubular maximal reabsorption of phosphate normalized to GFR
α-Klotho: Soluble alpha-Klotho

## References

[1] Foley RN, Parfrey PS, Sarnak MJ (1998). Epidemiology of cardiovascular disease in chronic renal disease. J Am Soc Nephrol.

[2] Olauson H, Vervloet MG, Cozzolino M, Massy ZA, Urena Torres P, Larsson TE (2014). New insights into the FGF23-klotho axis. Semin Nephrol.

[3] Kuro-o M, Matsumura Y, Aizawa H, Kawaguchi H, Suga T, Utsugi T, Ohyama Y, Kurabayashi M, Kaname T, Kume E (1997). Mutation of the mouse klotho gene leads to a syndrome resembling ageing. Nature.

[4] Hu MC, Shi M, Zhang J, Pastor J, Nakatani T, Lanske B, Razzaque MS, Rosenblatt KP, Baum MG, Kuro-o M, Moe OW (2010). Klotho: a novel phosphaturic substance acting as an autocrine enzyme in the renal proximal tubule. FASEB J.

[5] Kim HR, Nam BY, Kim DW, Kang MW, Han JH, Lee MJ, Shin DH, Doh FM, Koo HM, Ko KI (2013). Circulating alpha-klotho levels in CKD and relationship to progression. Am J Kidney Dis.

[6] Semba RD, Cappola AR, Sun K, Bandinelli S, Dalal M, Crasto C, Guralnik JM, Ferrucci L (2011). Plasma klotho and cardiovascular disease in adults. J Am Geriatr Soc.

[7] Kitagawa M, Sugiyama H, Morinaga H, Inoue T, Takiue K, Ogawa A, Yamanari T, Kikumoto Y, Uchida HA, Kitamura S (2013). A decreased level of serum soluble klotho is an independent biomarker associated with arterial stiffness in patients with chronic kidney disease. PLoS One.

[8] Hu MC, Shi M, Zhang J, Quinones H, Griffith C, Kuro-o M, Moe OW (2011). Klotho deficiency causes vascular calcification in chronic kidney disease. J Am Soc Nephrol.

[9] Saito Y, Nakamura T, Ohyama Y, Suzuki T, Iida A, Shiraki-Iida T, Kuro-o M, Nabeshima Y, Kurabayashi M, Nagai R (2000). In vivo klotho gene delivery protects against endothelial dysfunction in multiple risk factor syndrome. Biochem Biophys Res Commun.

[10] Li BS, Ma HX, Wang YJ, Wu P (2012). klotho gene attenuates the progression of hypertension and heart damage in spontaneous hypertensive rats. Zhonghua Yi Xue Yi Chuan Xue Za Zhi.

[11] Xie J, Yoon J, An SW, Kuro OM, Huang CL (2015). Soluble klotho protects against uremic cardiomyopathy independently of fibroblast growth factor 23 and phosphate. J Am Soc Nephrol.

[12] Ohta Junsuke, Rakugi Hiromi, Ishikawa Kazuhiko, Yang Jin, Ikushima Masashi, Chihara Yukana, Maekawa Yoshihiro, Oguro Ryosuke, Hanasaki Hiroko, Kida Iwao, Matsukawa Naomichi, Ogihara Toshio (2007). Klotho gene delivery suppresses oxidative stress in vivo. Geriatrics & Gerontology International.

[13] Mitani H, Ishizaka N, Aizawa T, Ohno M, Usui S, Suzuki T, Amaki T, Mori I, Nakamura Y, Sato M (2002). In vivo klotho gene transfer ameliorates angiotensin II-induced renal damage. Hypertension.

[14] Zhang H, Li Y, Fan Y, Wu J, Zhao B, Guan Y, Chien S, Wang N (2008). Klotho is a target gene of PPAR-gamma. Kidney Int.

[15] Yamagishi T, Saito Y, Nakamura T, Takeda S, Kanai H, Sumino H, Kuro-o M, Nabeshima Y, Kurabayashi M, Nagai R (2001). Troglitazone improves endothelial function and augments renal klotho mRNA expression in Otsuka long-Evans Tokushima fatty (OLETF) rats with multiple atherogenic risk factors. Hypertens Res.

[16] Yang HC, Deleuze S, Zuo Y, Potthoff SA, Ma LJ, Fogo AB (2009). The PPARgamma agonist pioglitazone ameliorates aging-related progressive renal injury. J Am Soc Nephrol.

[17] Saito K, Ishizaka N, Mitani H, Ohno M, Nagai R (2003). Iron chelation and a free radical scavenger suppress angiotensin II-induced downregulation of klotho, an anti-aging gene, in rat. FEBS Lett.

[18] Yoon HE, Ghee JY, Piao S, Song JH, Han DH, Kim S, Ohashi N, Kobori H, Kuro-o M, Yang CW (2011). Angiotensin II blockade upregulates the expression of klotho, the anti-ageing gene, in an experimental model of chronic cyclosporine nephropathy. Nephrol Dial Transplant.

[19] Mitobe M, Yoshida T, Sugiura H, Shirota S, Tsuchiya K, Nihei H (2005). Oxidative stress decreases klotho expression in a mouse kidney cell line. Nephron Exp Nephrol.

[20] Kuwahara N, Sasaki S, Kobara M, Nakata T, Tatsumi T, Irie H, Narumiya H, Hatta T, Takeda K, Matsubara H, Hushiki S (2008). HMG-CoA reductase inhibition improves anti-aging klotho protein expression and arteriosclerosis in rats with chronic inhibition of nitric oxide synthesis. Int J Cardiol.

[21] Karalliedde J, Maltese G, Hill B, Viberti G, Gnudi L (2013). Effect of renin-angiotensin system blockade on soluble klotho in patients with type 2 diabetes, systolic hypertension, and albuminuria. Clin J Am Soc Nephrol.

[22] Donate-Correa J, Henriquez-Palop F, Martin-Nunez E, Perez-Delgado N, Muros-de-Fuentes M, Mora-Fernandez C, Navarro-Gonzalez JF (2016). Effect of Paricalcitol on FGF-23 and klotho in kidney transplant recipients. Transplantation.

[23] Schmid C, Neidert MC, Tschopp O, Sze L, Bernays RL (2013). Growth hormone and klotho. J Endocrinol.

[24] Locher R, Egger A, Zwimpfer C, Sze L, Schmid C, Christ E (2015). Effect of growth hormone replacement therapy on soluble klotho in patients with growth hormone deficiency. Clin Endocrinol.

[25] Efthymiadou A, Kritikou D, Mantagos S, Chrysis D (2016). The effect of GH treatment on serum FGF23 and klotho in GH-deficient children. Eur J Endocrinol.

[26] Heijboer AC, Blankenstein MA, Hoenderop J, de Borst MH, Vervloet MG (2013). Laboratory aspects of circulating alpha-klotho. Nephrol Dial Transplant.

[27] Heijboer AC, Levitus M, Vervloet MG, Lips P, ter Wee PM, Dijstelbloem HM, Blankenstein MA (2009). Determination of fibroblast growth factor 23. Ann Clin Biochem.

[28] Walton RJ, Bijvoet OL (1975). Nomogram for derivation of renal threshold phosphate concentration. Lancet.

[29] Barker SL, Pastor J, Carranza D, Quinones H, Griffith C, Goetz R, Mohammadi M, Ye J, Zhang J, Hu MC (2015). The demonstration of alphaKlotho deficiency in human chronic kidney disease with a novel synthetic antibody. Nephrol Dial Transplant.

[30] Seiler S, Wen M, Roth HJ, Fehrenz M, Flugge F, Herath E, Weihrauch A, Fliser D, Heine GH (2013). Plasma klotho is not related to kidney function and does not predict adverse outcome in patients with chronic kidney disease. Kidney Int.

[31] Haruna Y, Kashihara N, Satoh M, Tomita N, Namikoshi T, Sasaki T, Fujimori T, Xie P, Kanwar YS (2007). Amelioration of progressive renal injury by genetic manipulation of klotho gene. Proc Natl Acad Sci U S A.

[32] Sugiura H, Yoshida T, Tsuchiya K, Mitobe M, Nishimura S, Shirota S, Akiba T, Nihei H (2005). Klotho reduces apoptosis in experimental ischaemic acute renal failure. Nephrol Dial Transplant.

[33] Sugiura H, Yoshida T, Mitobe M, Yoshida S, Shiohira S, Nitta K, Tsuchiya K (2010). Klotho reduces apoptosis in experimental ischaemic acute kidney injury via HSP-70. Nephrol Dial Transplant.

[34] Mak RH, Cheung WW, Roberts CT (2008). The growth hormone-insulin-like growth factor-I axis in chronic kidney disease. Growth Hormon IGF Res.

[35] Bach LA, Hale LJ (2015). Insulin-like growth factors and kidney disease. Am J Kidney Dis.

[36] Kopple JD, Cheung AK, Christiansen JS, Djurhuus CB, El Nahas M, Feldt-Rasmussen B, Mitch WE, Wanner C, Gothberg M, Ikizler TA (2011). OPPORTUNITY&trade;: a large-scale randomized clinical trial of growth hormone in hemodialysis patients. Nephrol Dial Transplant.

[37] Lindberg K, Amin R, Moe OW, Hu MC, Erben RG, Ostman Wernerson A, Lanske B, Olauson H, Larsson TE (2014). The kidney is the principal organ mediating klotho effects. J Am Soc Nephrol.

[38] Fischer DC, Nissel R, Puhlmann A, Mitzner A, Tiess M, Schmidt R, Haffner D (2009). Differential effects of short-term growth hormone therapy on the cardiovascular risk profile in patients with chronic kidney disease: a pilot study. Clin Nephrol.

[39] Nissel R, Fischer DC, Puhlmann A, Holdt-Lehmann B, Mitzner A, Petzsch M, Korber T, Tiess M, Schmidt R, Haffner D (2009). Short-term growth hormone treatment and microcirculation: effects in patients with chronic kidney disease. Microvasc Res.

[40] Gardner J, Ashraf A, You Z, McCormick K (2011). Changes in plasma FGF23 in growth hormone deficient children during rhGH therapy. J Pediatr Endocrinol Metab.

[41] Bianda T, Glatz Y, Bouillon R, Froesch ER, Schmid C (1998). Effects of short-term insulin-like growth factor-I (IGF-I) or growth hormone (GH) treatment on bone metabolism and on production of 1,25-dihydroxycholecalciferol in GH-deficient adults. J Clin Endocrinol Metab.

[42] Bianda T, Hussain MA, Glatz Y, Bouillon R, Froesch ER, Schmid C (1997). Effects of short-term insulin-like growth factor-I or growth hormone treatment on bone turnover, renal phosphate reabsorption and 1,25 dihydroxyvitamin D3 production in healthy man. J Intern Med.

[43] Kolek OI, Hines ER, Jones MD, LK LS, Lipko MA, Kiela PR, Collins JF, Haussler MR, Ghishan FK (2005). 1alpha,25-Dihydroxyvitamin D3 upregulates FGF23 Gene Expr in bone: the final link in a renal-gastrointestinal-skeletal axis that controls phosphate Transport. Am J Physiol Gastrointest Liver Physiol.

[44] Shimada T, Hasegawa H, Yamazaki Y, Muto T, Hino R, Takeuchi Y, Fujita T, Nakahara K, Fukumoto S, Yamashita T (2004). FGF-23 is a potent regulator of vitamin D metabolism and phosphate homeostasis. J Bone Miner Res.

[45] Saito H, Maeda A, Ohtomo S, Hirata M, Kusano K, Kato S, Ogata E, Segawa H, Miyamoto K, Fukushima N (2005). Circulating FGF-23 is regulated by 1alpha,25-dihydroxyvitamin D3 and phosphorus in vivo. J Biol Chem.

[46] Nishi H, Nii-Kono T, Nakanishi S, Yamazaki Y, Yamashita T, Fukumoto S, Ikeda K, Fujimori A, Fukagawa M (2005). Intravenous calcitriol therapy increases serum concentrations of fibroblast growth factor-23 in dialysis patients with secondary hyperparathyroidism. Nephron Clin Pract.

[47] Hansen D, Rasmussen K, Pedersen SM, Rasmussen LM, Brandi L (2012). Changes in fibroblast growth factor 23 during treatment of secondary hyperparathyroidism with alfacalcidol or paricalcitol. Nephrol Dial Transplant.

[48] Pedersen L, Pedersen SM, Brasen CL, Rasmussen LM (2013). Soluble serum klotho levels in healthy subjects. Comparison of two different immunoassays. Clin Biochem.

